# Vaginoscopic resection of hemivagina, in a 20‐year‐old virgin female with prior misdiagnosis of OHVIRA syndrome as a bicornuate uterus: A case report

**DOI:** 10.1002/ccr3.8661

**Published:** 2024-03-14

**Authors:** Ameneh Haghgoo, Ali Faegh, Saeed Nasiri, Farnaz Akhbari

**Affiliations:** ^1^ School of Medicine Iran University of Medical Sciences Tehran Iran; ^2^ School of Medicine Alborz University of Medical Sciences Karaj Iran; ^3^ Department of Radiology Imam‐Hossein Medical and Educational Center, Shahid Beheshti University of Medical Sciences Tehran Iran; ^4^ Department of Reproductive Biomedicine Research Royan Institute Tehran Iran

**Keywords:** bicornuate uterus, Herlyn–Werner–Wunderlich syndrome, hymen preservation, OHVIRA syndrome, vaginoscopy

## Abstract

**Key Clinical Message:**

OHVIRA syndrome can be misdiagnosed due to its rarity, resulting in the need for more invasive interventions than vaginoscopy. Also, delayed diagnosis of OHVIRA syndrome can affect patient's quality of life by leading to chronic gynecological diseases such as endometriosis and pelvic inflammatory disease.

**Abstract:**

Obstructive hemivagina and ipsilateral renal agenesis (OHVIRA) syndrome is one of the infrequent congenital Mullerian duct anomalies characterized by obstructed hemivagina and ipsilateral renal agenesis. This study presents a 20‐year‐old virgin female who was diagnosed with OHVIRA syndrome and treated by vaginoscopy using the hymen preservation technique. Also, she was misdiagnosed with non‐communicating rudimentary uterine horn 4 years ago. Late or misdiagnosis of OHVIRA syndrome can affect fertility and pregnancy outcomes. Therefore, early diagnosis and management are crucial. OHVIRA syndrome's misdiagnosis is possible with other Mullerian duct anomalies, such as a rudimentary uterine horn. Also, patients with misdiagnosis undergo unnecessary interventions.

## 1 INTRODUCTION

Obstructed hemivagina and ipsilateral renal agenesis (OHVIRA) syndrome is one of the infrequent Mullerian duct anomalies. OHVIRA syndrome arises from abnormal development of the Mullerian and Wolffian ducts.^1^ It is a congenital and urogenital anomaly, including uterine didelphys, obstructed hemivagina, and unilateral renal agenesis.^2^ The mean age of OHVIRA syndrome diagnosis is about 14 years. Also, adolescents are usually diagnosed with OHVIRA syndrome 1–2 years after menarche due to severe dysmenorrhea.^3^, ^4^ Severe lower abdominal pain with catamenial exacerbation is the most common clinical manifestation of OHVIRA syndrome; nevertheless, patients may be asymptomatic.^2^ In this study, we present a 20‐year‐old virgin female who was misdiagnosed with rudimentary uterine horn 4 years ago and diagnosed with OHVIRA syndrome when she presented to the author's office. This study is essential in two ways: (1) misdiagnosis of OHVIRA syndrome affects fertility outcomes, and (2) hymen was preserved in this patient using a rigid resectoscope.

## 2 CASE PRESENTATION (HISTORY AND EXAMINATION)

A 20‐year‐old virgin female presented to the author's office (Tehran‐Iran) in 2023 with the chief complaint of severe lower abdominal pain (Visual Analog Scale [VAS] = 10) that was exacerbated by menstruation during the past 4 years. She also had dysmenorrhea (VAS = 10) and dyschezia (VAS = 5). She attained menarche at the age of 13. The menstrual cycle duration was over 35 days. Also, she noticed an average amount of menstrual bleeding. She had not experienced vaginal intercourse until presenting. Also, she had no history of anemia and blood transfusion, and the hemoglobin level was within the normal range.

**FIGURE 1:**
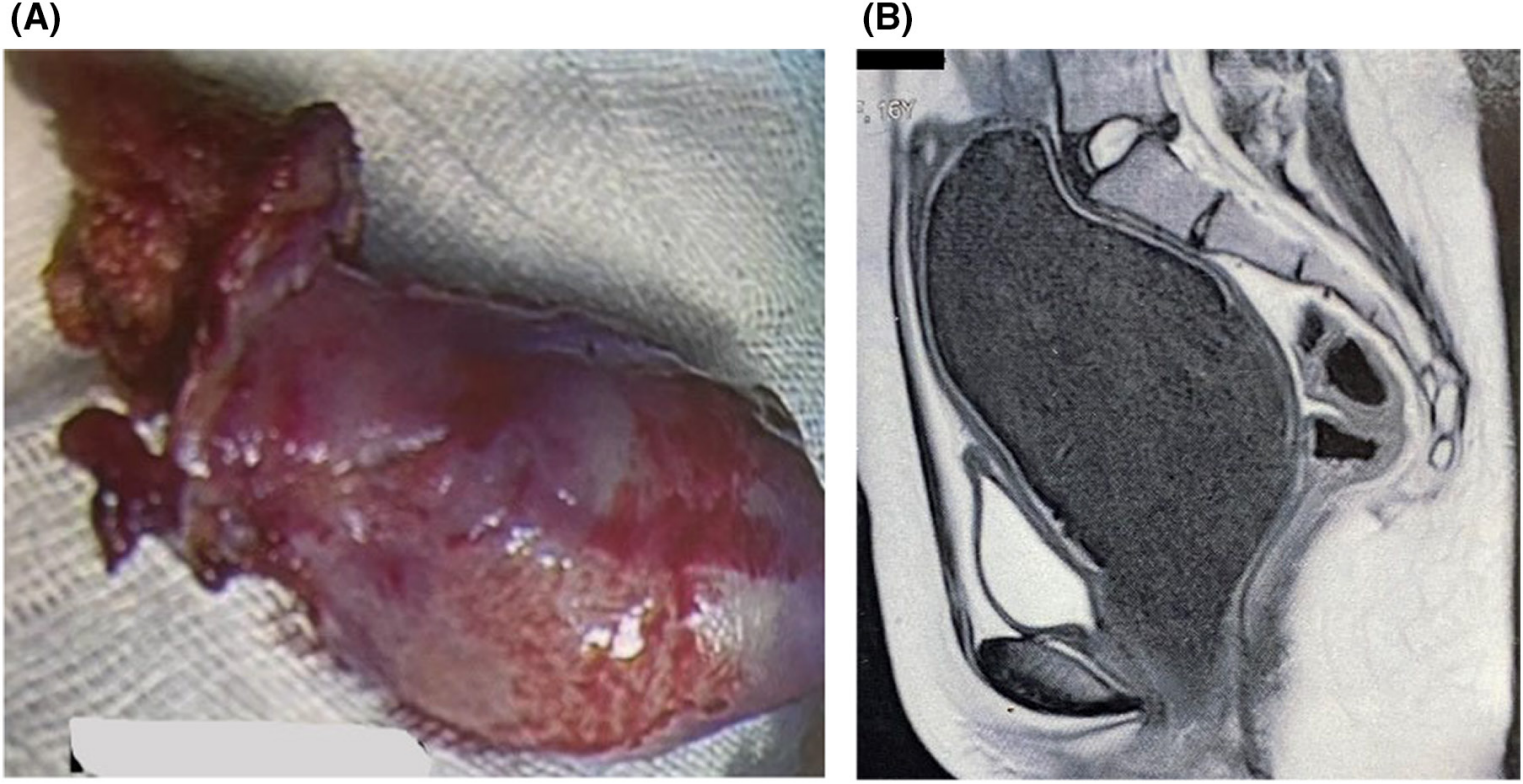
Patient's imaging findings and surgical interventions 4 years before presenting to us. (A) Rudimentary uterine horn resection; (B) hematocolpos revealed by magnetic resonance imaging (MRI).

Four years ago, she underwent laparoscopic uterine horn resection by a gynecologist according to the rudimentary uterine horn diagnosis (Figure 1). As shown in Figure 1, magnetic resonance imaging (MRI) demonstrated right kidney agenesis and hematocolpos before the surgery. However, she mentioned no improvement in her symptoms after surgery.

During the physical examination, she had lower abdominal tenderness. The patient was a virgin; therefore, the pelvic examination was not performed.

## 3 METHODS

**FIGURE 2:**
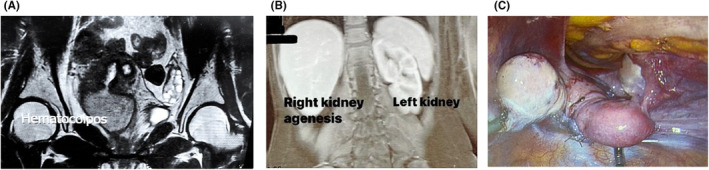
Imaging and surgical findings presented to us. (A) Hematocolpos; (B) right kidney agenesis; (C) Laparoscopic view of the uterine.

As illustrated in Figure 2, abdominal and pelvic MRI and ultrasound revealed right kidney agenesis, left kidney hypertrophy, didelphys uterus, remaining right cervix and uterus isthmus, right hemivagina transverse septum, and hematocolpos.

According to the patient's symptoms, history, and imaging assessments, she was diagnosed with OHVIRA syndrome (classification 1.1). Also, according to the severe dysmenorrhea, she was prescribed Mefenamic acid (250 mg, q6hr) until intervention.

She underwent vaginoscopy and vagina septum resection. An incision was made on the vaginal septum to drain the hematocolpos. The vaginal septum was resected, and about 500 cc of blood and black clots was drained. According to the patient's preference and cultural issues in her country region, we tried to keep the hymen intact as much as possible by using a resectoscope. The vaginoscopy video is available in a File S1.

Due to the high prevalence of endometriosis in patients with OHVIRA syndrome (about 30%), laparoscopic abdominal and pelvic exploration for endometriosis was performed^5^. Adhesiolysis was performed, and she didn't have any endometriosis implantations or nodules (Figure 2). Following the surgery, she received intravenous cephalothin and metronidazole, which were later switched to oral metronidazole and cephalexin for 3 days.

## 4 CONCLUSION AND RESULTS

At the 12‐month follow‐up, she reported substantial relief from dysmenorrhea and abdominal pain, as well as normal menstrual cycles. The follow‐up transabdominal ultrasound showed no signs of hematocolpos.

## 5 DISCUSSION

“Herlyn–Werner–Wunderlich syndrome” (OHVIRA syndrome) was first described by Purslow in 1922, and it is one of the extremely rare Mullerian duct anomalies.^6^ It is characterized by the triad of uterine didelphys, obstructed hemi‐ vagina, and ipsilateral renal agenesis.^3^, ^4^ Uterine didelphys occurs because of incomplete fusion of the Mullerian duct in the ninth week of gestational age.^7^ OHVIRA syndrome is usually diagnosed in adolescence (about 3–36 months after menarche), followed by severe lower abdominal pain with exacerbation during menstruation, dysmenorrhea, and sensing a paravaginal mass.^2^, ^4^ A visible bulged vaginal sidewall is the most common finding on examination.^2^ OHVIRA syndrome has two classifications: classification 1: completely obstructed hemivagina (1.1, with blind hemivagina; 1.2, cervicovaginal atresia without communicating uteri) and classification 2: incompletely obstructed hemivagina (2.1, partial resorption of the vaginal septum; 2.2, with communicating uteri).^8^

Incomplete vaginal obstruction (classification 2) can be asymptomatic.^2^ Therefore, delayed diagnosis can occur in these cases, leading to chronic gynecological diseases such as endometriosis and pelvic inflammatory disease.^9^ So, OHVIRA syndrome can coexist with endometriosis in about 30% of patients and pelvic inflammatory disease.^10^ Delayed diagnosis and treatment can influence pregnancy outcomes.^10^ However, the pregnancy rate in patients with OHVIRA syndrome is about 87%, and the live birth rate is 77%.

Abdominal and pelvic ultrasound imaging is the first line of imaging for diagnosis, and MRI can confirm the diagnosis after finding suspected signs in ultrasound.^2^ According to the rarity of OHVIRA syndrome, it is crucial to avoid misdiagnosis. As our patient, patients with OHVIRA syndrome can be misdiagnosed with other Mullerian duct abnormalities, such as the rudimentary horn of the bicornuate uterus.

The standard treatment is a transvaginal incision in the vaginal septum to drain the hematocolpos, followed by the complete excision of the septum by vaginoscopy. Nevertheless, a few cases with more complicated anatomical variations need laparoscopic interventions.^2^ Post‐surgical recurrence is uncommon, but incomplete resection of the vaginal septum and only making an incision on the vaginal septum can lead to re‐infusion, so few instances, experience hemivagina re‐obstruction after surgery. Therefore, few patients need additional surgical interventions.^3^ According to the hymen sensitivity, it is at risk of damage during the vaginoscopy. On the other hand, in some populations, cultural issues lead to patient's and their parents' preferences for virginity preservation. Therefore, as shown in a Video S1, using a rigid resectoscope is helpful.^5^, ^11^

In conclusion, OHVIRA syndrome can be misdiagnosed due to its rarity, resulting in the need for more invasive interventions than vaginoscopy. Unnecessary and invasive interventions can lead to uterine damage and affect patient's fertility and pregnancy outcomes. Delayed diagnosis of OHVIRA syndrome can affect patient's quality of life by leading to chronic gynecological diseases such as endometriosis and pelvic inflammatory disease.

## AUTHOR CONTRIBUTIONS

**Ameneh Haghgoo:** Conceptualization; investigation; methodology; supervision; writing – review and editing. **Ali Faegh:** Writing – original draft. **Saeed Nasiri:** Visualization; writing – review and editing. **Farnaz Akhbari:** Data curation; visualization; writing – review and editing.

## FUNDING INFORMATION

This research received no specific grant from funding agencies in the public, commercial, or not‐for‐profit sectors.

## CONFLICT OF INTEREST STATEMENT

The authors have no relevant financial or non‐financial interests to disclose.

## ETHICS STATEMENT

Nikan Hospital's ethics committee (Tehran, Iran) approved this study.

## CONSENT

The authors verify that a signed consent has been obtained from the patient to participate in the study and publish figures and a video of the procedure.

## Supporting information


Video S1.

## Data Availability

Additional data on this manuscript are available by contacting the corresponding author.
